# Application of optically-induced-dielectrophoresis in microfluidic system for purification of circulating tumour cells for gene expression analysis- Cancer cell line model

**DOI:** 10.1038/srep32851

**Published:** 2016-09-09

**Authors:** Tzu-Keng Chiu, Wen-Pin Chou, Song-Bin Huang, Hung-Ming Wang, Yung-Chang Lin, Chia-Hsun Hsieh, Min-Hsien Wu

**Affiliations:** 1 Department of Chemical and Materials Engineering, Chang Gung University, Taoyuan City, 33302, Taiwan (R. O. C.); 2 Graduate Institute of Biochemical and Biomedical Engineering, Chang Gung University, Taoyuan City, 33302, Taiwan (R. O. C.); 3 Division of Haematology/Oncology, Department of Internal Medicine, Chang Gung Memorial Hospital, Taoyuan City, 33302, Taiwan (R. O. C.); 4 Department of Chemical Engineering, Ming Chi University of Technology, Taiwan (R. O. C.)

## Abstract

Circulating tumour cells (CTCs) in a blood circulation system are associated with cancer metastasis. The analysis of the drug-resistance gene expression of cancer patients’ CTCs holds promise for selecting a more effective therapeutic regimen for an individual patient. However, the current CTC isolation schemes might not be able to harvest CTCs with sufficiently high purity for such applications. To address this issue, this study proposed to integrate the techniques of optically induced dielectrophoretic (ODEP) force-based cell manipulation and fluorescent microscopic imaging in a microfluidic system to further purify CTCs after the conventional CTC isolation methods. In this study, the microfluidic system was developed, and its optimal operating conditions and performance for CTC isolation were evaluated. The results revealed that the presented system was able to isolate CTCs with cell purity as high as 100%, beyond what is possible using the previously existing techniques. In the analysis of CTC gene expression, therefore, this method could exclude the interference of leukocytes in a cell sample and accordingly contribute to higher analytical sensitivity, as demonstrated in this study. Overall, this study has presented an ODEP-based microfluidic system capable of simply and effectively isolating a specific cell species from a cell mixture.

Cancer metastasis is the main cause of cancer-derived death^1^. Circulating tumour cells (CTCs) are rare cancer cell species present in the peripheral blood and have been documented since 1869^2^. The existence of CTCs in a blood circulation system is proven to be responsible for cancer metastasis or relapse^1^. In cancer treatments, therefore, the CTCs in the blood circulation are regarded as an important chemotherapeutic target^3^. More recent literature reports have revealed that the chemotherapeutic drug resistances of the CTCs from epithelial cancers can be evaluated through the gene expression analysis of the drug transporters or so-called multi-drug-resistance-related proteins (MRPs)^4^,^5^ of CTCs. For the latter, several studies have reported that the expression levels of MRPs^6^, ALDH1^4^, ERCC-1^7^, CD133^8^, and thymidylate synthase^9^ in CTCs are predictive of resistance to chemotherapy^6^. Through analysing the anticancer drug-resistance gene expression of a patient’s CTCs, overall, a more effective therapeutic regimen can be selected for an individual patient to achieve so-called personalized cancer chemotherapy^10^.

To achieve the goal mentioned above, it is necessary to isolate and purify the CTCs from a blood sample with a certain quality requirement (i.e., high CTC purity). However, CTCs are very rare in a blood sample, with an approximate concentration of 1 CTC per 10^5^–10^7^ blood mononuclear cells^11^. This rarity makes them technically demanding to isolate and purify. With the recent progress in cell isolation and separation techniques, a wide variety of CTC isolation strategies have been actively proposed, which can be generally categorized into physical and biochemical methods^12^. Among the biochemical techniques, immunomagnetic separation approaches are predominantly utilized for these tasks. In these methods, magnetic beads coupled with CTC surface antigen [mainly the epithelial cell adhesion molecule (EpCAM) and cytokeratins (CKs)]-specific antibodies are commonly used to recognize and bind the CTCs^13^. The magnetic bead-bound CTCs are then separated from the leukocytes via an applied magnetic field. Cell isolation based on this strategy is usually referred to as positive selection of CTCs, primarily utilized in current CTC isolation and detection [e.g., the CellSearch^TM^ system^14^ or the magnetic-activated cell sorting system (MACS^®^)]^15^.

Borrowing from the technical merits of microfluidic technology, moreover, several microfluidic systems have been proposed for the isolation of CTCs with superior performance compared to the conventional macro-scale devices^16^,^17^. For example, the CTC-iChip^18^, lateral magnetophoresis chip^19^, two-stage microfluidic chip^20^, nanostructure embedded microchips^21^, parallel flow micro-aperture chip^22^, and the herringbone chip^23^ mainly utilize EpCAM- or other surface antigen-specific antibodies to recognize and capture CTCs in the microfluidic systems. Overall, these systems have been proven effective to isolate CTCs with both high CTC purity (14–70%)^18^,^20^,^23^ and high recovery rate (77–91.8%)^18^,^21^,^23^.

Although the abovementioned positive selection-based CTC isolation schemes (either the conventional- or microfluidic-based methods) have been technically proven effective to isolate and purify CTCs, there are some important biological issues that should be further considered. As discussed earlier, the majority of CTC isolation or purification strategies rely primarily on the use of EpCAM or CKs for the identification of CTCs. Nevertheless, EpCAM and CKs are not expressed in all tumours (e.g., sarcoma or melanoma)^24^, and thus, some kinds of CTCs might not be harvested through the positive selection-based CTC isolation schemes. Moreover, the CTCs, particularly ones with a highly metastatic nature, might undergo a so-called epithelial-to-mesenchymal transition (EMT)^25^. After that, the CTCs might down-regulate the expression of EpCAM and CKs^24^ and become motile cells for migration to distant metastatic sites^26^. Conversely, it has also been reported that non-epithelial cells (e.g., leukocytes) can express epithelial biomarkers such as EpCAM and CKs^27^. Taken together, all these phenomena could lead to biased outcomes of CTC isolation, and more importantly, the clinically meaningful CTCs associated with cancer metastasis might be missed.

To obtain the all possible CTCs in a blood sample for the subsequent biological assays, more recently, negative selection-based schemes for CTC isolation have been proposed^28^. In these methods, only blood cells are targeted for depletion, leaving behind all possible CTCs in the sample. Although this cell isolation strategy holds great promise for harvesting all possible CTCs for subsequent assays, most of these methods reported in the literature commonly suffered from lower purity of CTC isolation (10–16%)^29^,^30^ compared with their positive selection-based CTC isolation counterparts (62–70%)^16^,^18^,^31^. The low CTC purity issue in negative (or even positive) selection-based CTC isolation schemes could in turn hinder the subsequent utilization of CTCs for the analysis of anticancer drug-resistance gene expression.

To address this critical technical hurdle, we proposed to integrate the technique of optically induced dielectrophoretic (ODEP) force-based cell manipulation in a microfluidic system to further purify CTCs after a conventional CTC isolation process. In this study, the microfluidic system with the ODEP mechanism integrated was designed and fabricated. Moreover, the appropriate width of the side microchannel, working flow rate for sample transportation, and optimal ODEP operating conditions for the isolation of CTCs were experimentally determined. The CTC purification performance based on the presented method was evaluated. Finally, the impact of cancer cell purity on the analysis of anticancer drug-resistance gene expression was experimentally investigated. The results revealed that the presented ODEP-based microfluidic system was capable of isolating CTCs with cell purity as high as 100% (cell recovery rate: 41.5%). In the analysis of the anticancer drug-resistance gene expression levels of CTCs, therefore, this method could potentially exclude the interference of leukocytes in the cell samples tested and accordingly contribute to higher analytical sensitivity.

## Results and Discussions

### Technical features and design of the ODEP-based microfluidic system for CTC isolation

**Figure 1:**
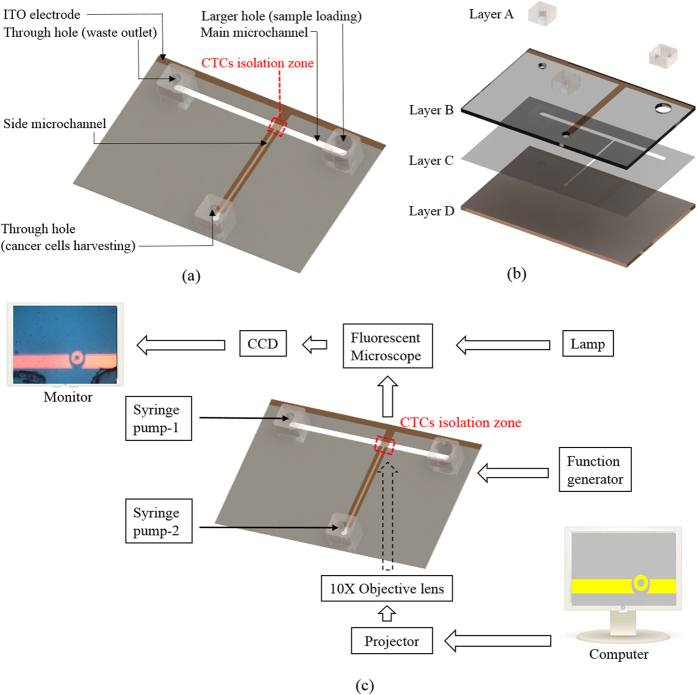
Schematic illustration of the (**a**) top-view layout and (**b**) assembly of the microfluidic system (Layer A: PDMS; Layer B: Etching-treated ITO glass; Layer C: Microfabricated adhesive tape; Layer D: ITO glass coated with photoconductive material) and the (**c**) overall experimental setup.

**Figure 2:**
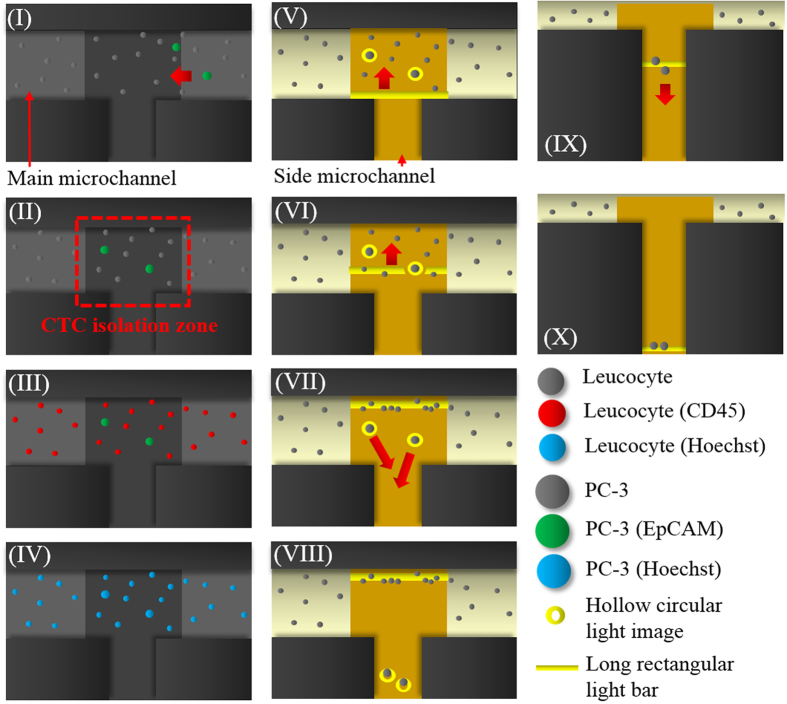
Schematic illustration of the overall cancer cell isolation processes (I) Fluorescent microscopic observation was performed in the CTC isolation zone to detect the cancer cell images (green dots) in a dynamic cell suspension flow; (II) the cell suspension flow was temporarily suspended when the cancer cells (green dots) were observed in the CTC isolation zone; (III) fluorescent microscopy operations were performed to observe the leukocytes (red dots), cancer cells (green dots) and (IV) all nucleated cells (blue dots); (V) the target cancer cells [i.e., EpCAM marker- and Hoechst dye-positive (green and blue dots) and CD45 marker-negative images (non-red dots)] were precisely positioned under light field microscopic imaging through the abovementioned contradistinction; (VI) ODEP force-based cell manipulation was performed to separate the cancer cells targeted from the leukocytes: hollow circular light images (ID: 40 μm; light bandwidth: 40 μm) were used to enclose the target cancer cells, and a long rectangular light bar (bar length: 1000 μm; bar width: 150 μm) was used to manipulate the leukocytes; (VII) the long rectangular light bar was moved (moving velocity: 100 μm s^−1^) to sweep all unenclosed leukocytes to the one side of the main microchannel leaving the enclosed cancer cells in the same positions; (VIII) the hollow circular light images were moved (moving velocity: 50 μm s^−1^) to manipulate the enclosed cancer cells to the side microchannel for collection; (IX)-(X) another moving rectangular light bar (bar length: 1000 μm; bar width: 150 μm; moving velocity: 100 μm s^−1^) was used to transport the cancer cells collected to a site near the through-hole for harvesting.

In this study, the combination of ODEP force-based cell manipulation and laminar flow in a microfluidic system was established (Fig. 1) for CTC isolation based on the working scheme described in Fig. 2. With the recent progress in Bio-MEMS (Bio-Micro-Electro-Mechanical System) technology, a wide variety of novel approaches such as acoustophoresis^32^, magnetophoresis^33^, thermophoresis^34^, dielectrophoresis (DEP)^29^, and optically induced-dielectrophoresis (ODEP)^35^ have been proposed for the manipulation of biological substances (e.g., cells^36^, bacteria^37^, or DNA^38^). Among them, DEP force-based cell manipulation has been widely adopted for various applications^29^,^39^. However, it normally requires a costly, time-consuming, and technically demanding microfabrication process to create a unique metal electrode layout that is specific to the application. The key technical merit of the ODEP force-based technique is that it can easily and quickly create or modify an electrode layout through the control of optical patterns, acting as a virtual electrode^40^. In operation, one can simply use a commercial digital projector to display optical images on the ODEP system to manipulate cells in a simple, flexible and user-friendly manner through a computer-interfaced control^36^. In this study, moreover, the inherent nature of laminar flow in a microfluidic system was used to transport a cell suspension in the main microchannel (Fig. 2). This allowed the biological cells to be delivered unidirectionally without cross-contamination of the sample caused by fluidic turbulent flow. Furthermore, immunofluorescent microscopic observation was conducted in the CTC isolation zone to specifically identify and position the target cancer cells. This step was followed by implementing ODEP force to precisely separate the cells of interest from the cell mixture, as described in Fig. 2. Through the specific biochemical differentiation of cell identity, CTC isolation with higher cell purity can be achieved.

**Figure 3:**
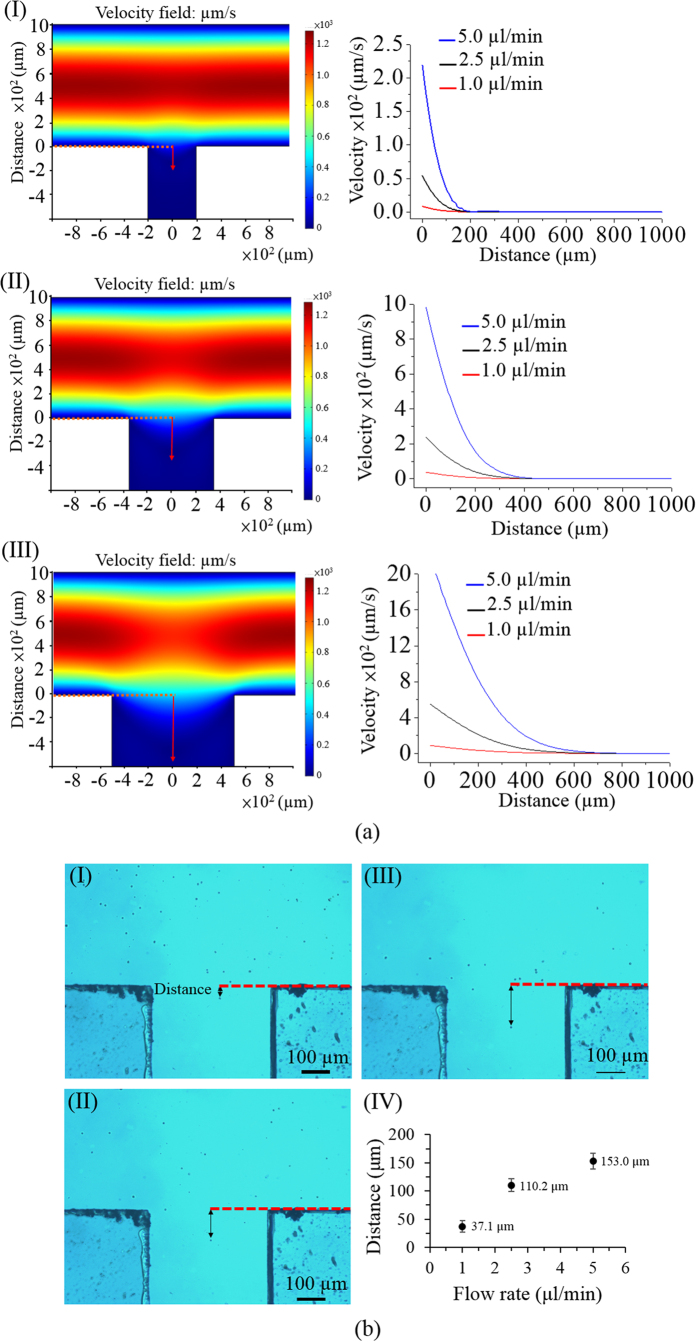
(**a**) Simulated flow patterns of T-shaped microchannel with three different side microchannel widths [(I) 400, (II) 700, and (III) 1,000 μm] under a given flow rate of 1 μl min^−1^ in the main microchannel (the left column); simulated flow velocity profiles along with the distance from the central start point of the side microchannel under three different side microchannel widths [(I) 400, (II) 700, and (III) 1,000 μm] and three different flow rate conditions (1.0, 2.5, and 5.0 μl min^−1^) in the main microchannel (the right column); (**b**) microscopic observations of the travel distance of a cell from the main to the side microchannel under three different flow rate conditions [(I) 1.0, (II) 2.5, and (III) 5.0 μl min^−1^)] in the main microchannel (given side microchannel width: 400 μm); (IV) the quantitative relationship between the measured travel distance of a cell from the main to side microchannel and the flow rate conditions (1.0, 2.5, and 5.0 μl min^−1^) in the main microchannel.

In terms of design, a simple microfluidic system with a T-shaped microchannel was fabricated [Fig. 1(a)]. As described in Fig. 2, a cell suspension was transported through the main microchannel, during which the cancer cells were identified and then selectively delivered to the side microchannel for collection. To avoid contamination from the leukocytes in the sample flow to the side microchannel, CFD-based simulations and experimental validations were conducted. This was to determine the appropriate width of the side microchannel and working flow rate of the cell suspension flow. Figure 3(a) (the left column) illustrates the simulated flow patterns of a T-shaped microchannel with three different side microchannel widths [(I) 400, (II) 700, and (III) 1,000 μm] under a given cell suspension flow rate of 1 μl min^−1^ in the main microchannel. To further evaluate the fluidic interference to the side microchannel, Fig. 3(a) (the right column) quantitatively shows the flow velocity profiles along with the distance from the central starting point of the side microchannel for three different side microchannel widths [(I) 400, (II) 700, and (III) 1,000 μm] and three different sample flow rate conditions (1.0, 2.5, and 5.0 μl min^−1^) in the main microchannel. Overall, it can be clearly seen from Fig. 3(a) that the narrower side microchannel or lower sample flow rate in the main microchannel resulted in less fluidic interference to the side microchannel under the conditions compared. Therefore, the proposed microfluidic system with a side microchannel width of 400 μm was designed. At a given side microchannel width of 400 μm, the sample flow rate issue discussed above was further experimentally validated as shown in Fig. 3(b). The travel distance of a cell from the main to side microchannel increased with the increasing sample flow rate in the main microchannel. In the results, the abovementioned travel distances were measured to be 37.1 ± 10.7, 110.2 ± 11.6, and 153.0 ± 13.7 μm under three different sample flow rate conditions (1.0, 2.5, and 5.0 μl min^−1^, respectively) in the main microchannel. For the simulation counterpart [Fig. 3(a–I) the right figure], the distances (from the central starting point of the side microchannel) where the flow velocity nearly reached zero were 40.2, 125.6, and 170.9 μm for the sample flow rate conditions of 1.0, 2.5, and 5.0 μl min^−1^, respectively. As a whole, the experimental results [Fig. 3(b)] correspond well to the simulation results [Fig. 3(a–I) the right figure]. Based on the fundamental investigations, therefore, a 2.5 μl min^−1^ sample flow rate was selected for operation as a compromise between the fluidic interference issue and working throughput.

### Operating conditions for ODEP force-based cell manipulation

In this study, ODEP force-based cell manipulation was conducted in the microfluidic system to separate the cancer cells from the leukocytes based on the guidance of immunofluorescent imaging (Fig. 2). For this purpose, the working conditions of the ODEP force for manipulating these cells were first characterized. In this evaluation, instead of ODEP force, the ODEP manipulation forces generated on the PC-3 cells and leukocytes were experimentally investigated. This is because the external forces acting on a manipulated cell in a real situation are complex. Within the external forces, the friction force is considered to play an important role^35^. Under a positive ODEP force, for example, a manipulated cell is attracted to the surface of an amorphous silicon layer. When a light image is moved to pull the cell, the friction force between the cell and surface should not be neglected. To simplify the forces exerting on a cell, therefore, the ODEP manipulation force, a net force between the ODEP force and friction force, has been commonly used in previous studies^35^,^36^.

Stokes’ law describes the drag force (*F*) exerted on a spherical particle (e.g., a cell) in a continuous flow condition and can be expressed as equation 1:


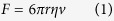


where *r*, *η*, and *v* denote the radius of a cell, the viscosity of the fluid, and the terminal velocity of a cell, respectively. According to Stokes’ law (equation 1), the manipulation force acting on a cell can be assumed to be equal to the hydrodynamic drag force exerting on a moving cell^35^. As a result, the manipulation force can therefore be experimentally estimated through the maximum manipulation velocity of a moving light image that can manipulate a cell, as discussed previously^35^,^36^.

In theory, the ODEP force generated on a cell can be expressed by equation 2 describing DEP force^41^:


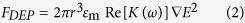


where *r*, *ε*_*m*_, and ∇*E*^*2*^ denote the cell radius, the permittivity of the solution surrounding the cells, and the gradient of the electrical field squared, respectively. *K*(*ω*), the Clausius–Mossotti factor, is further related to the angular frequency of the electric field, the conductivity of the medium, the internal conductivity of the cells, and the membrane capacitance of the cells^42^. Taken together, according to equation (2), the ODEP force generated on a cell is related to the inherent nature of the cell (e.g., size, conductivity, permittivity, or membrane capacitance) and the extrinsic conditions (e.g., magnitude and frequency of the electric voltage applied or the conductivity of the medium solution around cells). In terms of operating conditions, therefore, the ODEP force generated on a specific cell is dependent on the magnitude of the electric voltage applied at the given solution property conditions and the frequency of the electric voltage applied.

In this study, experimental investigations were performed to determine the quantitative link between the manipulation forces and the magnitude of electric voltage applied. For this purpose, the maximum velocity of a moving light image that could manipulate (e.g., drag or push) a cell was experimentally measured to calculate the manipulation force based on Stokes’ law (equation 1) as previously described^35^. To effectively separate the cancer cells from the leukocytes in this study, moreover, the operating conditions for the live and dead cells in the two cell species studied were further considered. This is because the ODEP forces acting on the live and dead cells could be different^43^. It was reported previously that the live and dead cells might experience positive and negative ODEP forces, respectively, under the same operating conditions^43^ in which the exerted ODEP force attracted the live cells but repelled the dead cells. In operation, a light image was used to simultaneously pull and push the live and dead cells, respectively. This phenomenon has been thoroughly discussed previously^43^ and is mainly due to the difference in cell membrane integrity between the live and dead cells. The integrity difference of the cell membrane could accordingly result in differences in the internal conductivity of cells (related to the Clausius–Mossotti factor, as previously mentioned) and in turn lead to the reverse response to an ODEP field^42^.

**Figure 4:**
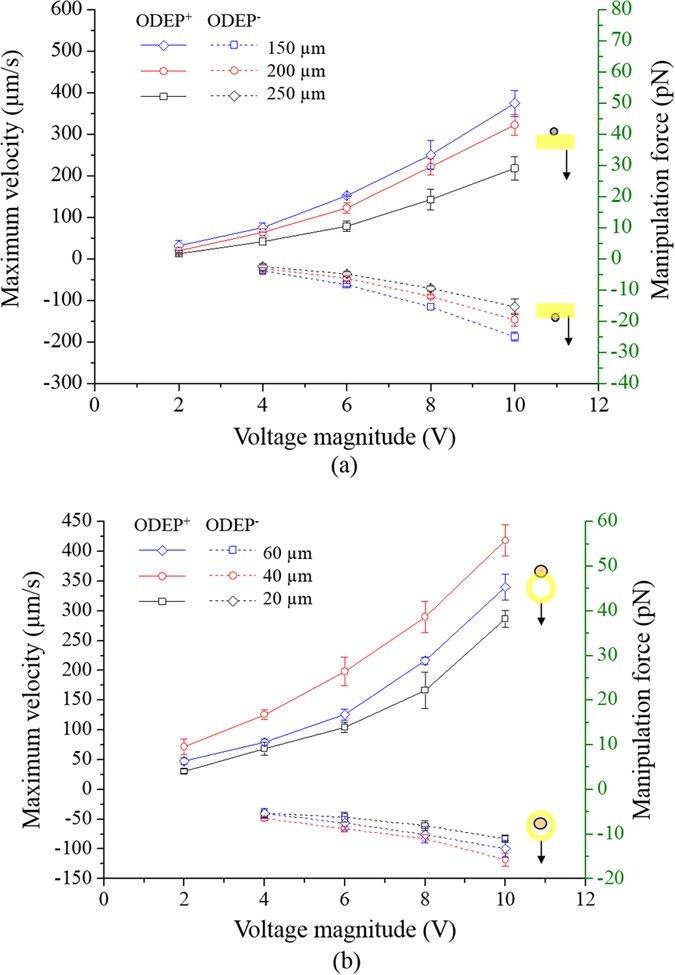
Evaluation of the maximum manipulation velocities and ODEP manipulation forces generated under different voltage and light image conditions: (**a**) voltage magnitude range: 2–10 V, bandwidth of long rectangular light bar: 150, 200, and 250 μm; (**b**) voltage magnitude 2–10 V, bandwidth of hollow circular light images (fixed ID: 40 μm): 20, 40, and 60 μm [results given as the mean ± standard deviation of 3 separate experiments (n = 9)]. (ODEP^+^: positive ODEP; ODEP^−^: negative ODEP force).

Figure 4(a) revealed the effect of the voltage magnitude applied (2–10 V) and the bandwidth of the light bar utilized (150, 200, and 250 μm) on the measured maximum velocities of the light bar (and the corresponding manipulation force calculated: right y axis) that can manipulate the live and dead leukocytes. For both a positive and negative ODEP (ODEP^+^ and ODEP^−^) force, the results in Fig. 4(a) corresponded well with equation (2), showing that the magnitude of the maximum manipulating velocity (or manipulation force) was proportional to the voltage squared. Although a higher magnitude of electric voltage could correspondingly generate a higher ODEP manipulation force for more efficient cell manipulation, ODEP force-based cell manipulation under a high electric field could lead to cell aggregation, which in turn might affect the cell separation process described in Fig. 2. This phenomenon was also observed and discussed in previous studies^36^. In this study, the magnitude of 8 V was used, which was a compromise between the manipulation force generated for cell manipulation and the cell aggregation phenomenon based on our microscopic observations. Regarding the influence of the bandwidth of the light bar on the ODEP manipulation forces, at a given voltage, the condition of 150 μm bandwidth was measured to have a statistically higher magnitude of maximum manipulating velocity (or manipulation force) than 250 μm bandwidth in the cases of both live and dead leukocytes [Fig. 4(a)]. This finding could be explained by the fact that, under an ODEP field, the electric field within a smaller light image could be more focused than in a larger one, accordingly contributing to a higher ODEP manipulation force^44^. Therefore, the light bar with 150 μm bandwidth was used for leukocyte manipulation in this work. After the magnitude of the electric voltage (8 V) and bandwidth of the light bar (150 μm) were determined, the maximum velocities of the moving light bar that can manipulate (pull) live and (push) dead leukocytes were measured to be 250.49 ± 34.63 μm/s and 115.57 ± 7.73 μm/s, respectively [Fig. 4(a)]. In the operation processes described in Fig. 2, a long moving rectangular light bar was utilized to sweep all the leukocytes to one side of the main microchannel for separation from the target cancer cells. For this purpose, the moving velocity of the light bar was set to 100 μm/s, allowing all the live and dead leukocytes to be simultaneously pulled and pushed, respectively, to one side of the main microchannel based on the above experimental evaluations.

For the live and dead cancer cells, hollow circular light images (fixed ID: 40 μm) were used to enclose and then manipulate the target cancer cells. As in the previously described evaluations [Fig. 4(a)], the influence of the voltage magnitude applied (2–10 V) and the bandwidth of the hollow circular light image utilized (20, 40, and 60 μm) on the measured maximum velocities of the light image (and the corresponding manipulation force calculated) that could manipulate the live and dead cancer cells were experimentally evaluated. Whether the cancer cells were alive or dead, overall, results [Fig. 4(b)] similar to the ones in Fig. 4(a) were found again in which the magnitudes of the maximum manipulating velocities were proportional to the applied voltage squared. In addition, the phenomenon of cell aggregation was clearly observed when the magnitude of the voltage applied was higher than 8 V. Therefore, the magnitude of electric voltage was set to 8 V for the ODEP-based cell manipulation in this study. Regarding the bandwidth (20, 40, and 60 μm) effect of the light images, Fig. 4(b) shows that the hollow circular light image (fixed ID: 40 μm) with 40 μm bandwidth had the highest ODEP manipulation force (and maximum manipulating velocity) compared with the other two cases explored (middle and lowest: 60 and 20 μm bandwidth, respectively). As in the above explanations, a light image with smaller size (e.g., 40 μm bandwidth) could lead to a higher ODEP force than one with a larger size (e.g., 60 μm bandwidth)^44^. Nevertheless, it was unexpected that the light image with the smallest bandwidth (e.g., 20 μm) had the lowest cell manipulation force (and maximum manipulating velocity) [Fig. 4(b)]. This finding could be because this bandwidth (20 μm) of the light image was close to the average size (estimated diameter: 23 ± 2.1 μm) of the cancer cells manipulated in this study, which could, in turn, result in instability of the ODEP force generation, as discovered previously^42^,^44^. Based on the above characterizations, overall, the magnitude of the electric voltage (8 V) and the bandwidth of the hollow circular light image (40 μm) were determined. Under these conditions, the maximum velocities of a moving hollow circular light image that can manipulate (pull) the live and (push) dead cancer cells were measured to be 290.01 ± 26.33 μm/s and 82.84 ± 7.37 μm/s, respectively [Fig. 4(b)]. Based on these results, the moving velocity of the hollow circular light image was set at 50 μm/s to ensure that the hollow circular light images could simultaneously manipulate the live (pull) and dead (push) cancer cells during operation [Fig. 2 (VII)-(VIII)].

### CTC purification performance using the ODEP-based microfluidic system

**Figure 5:**
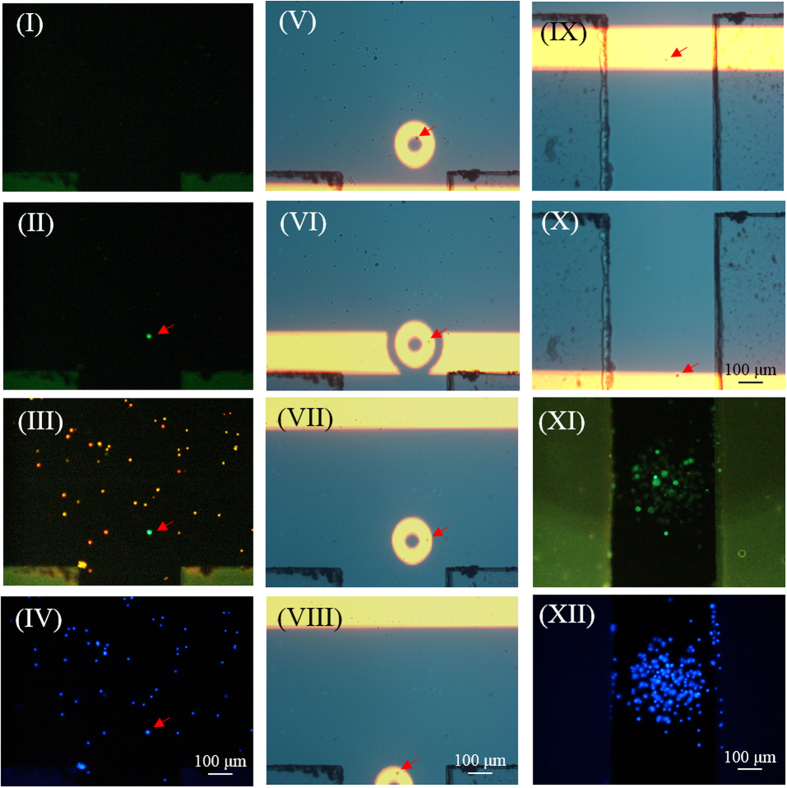
The overall CTC isolation and purification processes: (I) fluorescent microscopic observation was performed on the CTC isolation zone to detect the cancer cell images (green dots) in a dynamic cell suspension flow; (II) the cell suspension flow was temporarily suspended when the cancer cells (green dots) were observed in the CTC isolation zone; (III)-(IV) fluorescent microscopy operations were performed to observe the leukocytes (red dots), cancer cells (green dots), and all nucleated cells (blue dots) for cancer cell positioning purposes; (V) a hollow circular light image was used to enclose the target cancer cells, and a long rectangular light bar was used to manipulate the leukocytes; (VI)-(VII) the long rectangular light bar was moved to sweep all unenclosed leukocytes to one side of the main microchannel, leaving the enclosed cancer cells in the same positions; (VIII) the circular light image was moved to manipulate enclosed the cancer cells to the side microchannel for collection; (IX)-(X) another moving rectangular light bar was used to transport the cancer cells collected to a site near the through-hole for harvesting; (XI)-(XII) immunofluorescent microscopic observations were performed to examine the purity of cancer cells [the leukocytes (red dots), cancer cells (green dots), and all nucleated cells (blue dots)]. (Three video clips are provided as the 1^st^, 2^nd^ and 3^rd^ video clips).

To test the performance of the presented method for CTC isolation and purification, a cell suspension sample was prepared by spiking PC-3 cells (500 cells) into a whole blood sample (8 ml), followed by a negative selection-based CTC isolation process. The cell pellets obtained were stained with immunofluorescent dyes, and then re-suspended in 30 μl of sucrose solution. The treated cell suspension sample was subsequently loaded into the microfluidic system for further CTC isolation and purification based on the working scheme described in Fig. 2. Figure 5 shows the overall CTC isolation processes, from cancer cell identification and positioning using fluorescent microscopic observations [Fig. 5(I–IV)] to cancer cell separation via ODEP force-based cell manipulation with the aid of light field microscopic imaging [Fig. 5(V–X)]. By repeating these processes, the cancer cells isolated and temporarily collected in the side microchannel were checked for cancer cell purity using fluorescent microscopic observation. Figure 5 (XI) and (XII) showed that the cells in the side microchannel were all EpCAM marker (green dots)- and Hoechst dye (blue dots)-positive cancer cells without the existence of CD45 marker (red dots)-positive leukocytes. Overall, the results demonstrated that the proposed method was capable of isolating cancer cells with cell purity as high as 100%, which is currently impossible using existing CTC isolation techniques. Moreover, the recovery rate of cancer cells, and minimum number of cancer cells per millilitre of blood that can be isolated were experimentally evaluated to be 41.5%, and 15 cells ml^−1^, respectively. In addition to the abovementioned cancer cell line model tested, the use of the proposed method for the isolation of real CTCs from a cancer patient’s blood was also successfully demonstrated. Result (Supplementary figure; Fig. S2) revealed that the presented method was able to harvest 100% pure real CTCs, in which no leukocyte [red dots: Fig. S2-(b)-(II)] was found in the cells harvested. In the future application, it can be reasonably believed that the recovery rate of target cells, and working throughput can be further improved through the integration of a programmable image analysis system, and automation mechanism in the proposed system.

### Effect of leukocyte contamination on the analysis of anticancer drug-resistance gene expression of cancer cells

**Figure 6:**
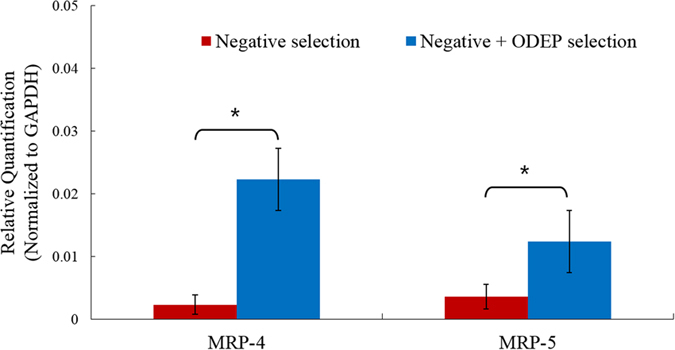
Comparison of relative gene (MRP4 and MRP5) expression levels of the cancer cells isolated by the two CTC isolation schemes (namely, a negative selection-based CTC isolation method and the same method with an additional ODEP-based CTC isolation process) studied. In the comparison, the relative quantification of the MRP4 and MRP5 gene expression levels of cancer cells was conducted. The gene expression levels were normalized to individual house-keeping genes (GAPDH) [results were given as the mean ± standard deviation of 3 separate experiments. Significant difference is indicated by stars (*p < 0.05, ANOVA)].

The analysis of CTCs’ anticancer drug-resistance gene expression holds great promise to predict the CTCs’ responses to chemotherapeutic drugs^6^. Such evaluation is found clinically useful for so-called personalized chemotherapy or precision medicine. In current CTC isolation methods, however, the contamination of leukocytes in the final products is commonly unavoidable. This could cause problems in the subsequent use of CTCs for the analysis of anticancer drug-resistance gene expression. This is primarily because the expression levels of the anticancer drug-resistance genes of leukocytes are unclear. Therefore, their presence could interfere with subsequent analytical work. To understand to what extent the analysis of cancer cells’ anticancer drug-resistance gene expression levels can be hampered by the existence of leukocytes in the sample obtained after a CTC isolation process, experimental evaluations were conducted. As a test model, PC-3 cells were spiked into a blood sample (62.5 cancer cells/ml blood). The sample was then treated with two kinds of CTC isolation protocols (namely a negative selection-based CTC isolation and the former method with an additional ODEP-based CTC isolation scheme, as described in Fig. 2). After CTC isolation, the cells obtained were subsequently analysed for gene expression (i.e., the mRNA levels of EpCAM, CD45, MRP4, MRP5 and GAPDH). First, the analysis of mRNA levels of EpCAM, and CD45 of the cells obtained through the two abovementioned CTC isolation protocols was to further compare the CTC purity in the cells harvested. The results revealed that the cells harvested from the ODEP-based CTC isolation protocol only detected the gene expression of EpCAM, a surface marker on cancer cells of epithelial origins, whereas no gene expression of the typical leukocyte surface marker CD45 was observed (Supplementary figure; Fig. S3). This result further supported that the ODEP-based CTC isolation scheme was able to isolate 100% pure CTCs after a negative selection-based CTC isolation process, as shown in Fig. 5(XI,XII). For the anticancer drug-resistance gene expression, moreover, the expression levels of two drug resistance genes (i.e., MRP4, and MRP5)^45^ were analysed as demonstration cases. The results (Fig. 6) showed that the expression levels of MRP4 and MRP5 drug resistance genes in the cell samples obtained from the ODEP-based CTC isolation protocol were significantly higher (*p *< 0.05) (MRP4 and MRP5: 9.69 and 3.44-fold higher, respectively) than the cases without further treatment using ODEP-based CTC isolation. In this study, the relative quantification of drug resistance gene expression levels of cancer cells was conducted in which the gene expression levels were normalized to individual house-keeping genes (GAPDH). Therefore, the lower expression levels of drug resistance genes found above could be due to the low CTC purity (Supplementary figure; Fig. S3) in the cell samples obtained from the CTC isolation treatments without further purification. In practical applications, therefore, the use of CTCs with low cell purity could underestimate the expression levels of drug resistance genes, as found in Fig. 6. In contrast, the ODEP-based CTC isolation method was able to obtain high-purity cancer cells, possibly excluding the interference of leukocytes in the cell samples tested. This exclusion could accordingly contribute to higher analytical sensitivity.

## Materials and Methods

### Design of the ODEP microfluidic system for CTC purification

The presented microfluidic system was designed to semi-continuously separate the cancer cells from a leukocyte background using the technique of ODEP force-based cell manipulation and the inherent laminar flow phenomenon in a microfluidic system. The layout of the microfluidic system is schematically shown in Fig. 1(a). In this work, a T-shaped microchannel was designed, in which the main microchannel (L: 25 mm, W: 1000 μm, H: 100 μm) was used for fresh and waste sample transportation, and the side microchannel (L: 15 mm, W: 400 μm, H: 100 μm) was used for cancer cell collection. In the design, the ODEP force-based cell manipulations for cancer cell isolation were performed at the junction area (L: 1000 μm, W: 1000 μm, H: 100 μm) of two microchannels, defined as the CTC isolation zone. In the microfluidic system [Fig. 1(a)], two through-holes (D: 1 mm) for tubing connections were designed, one for collecting the waste sample and the other for harvesting the isolated cancer cells. In addition, a larger hole (D: 2.5 mm) served as a reservoir for fresh sample loading [Fig. 1(a)]. The assembly of the microfluidic system is schematically illustrated in Fig. 1(b). Briefly, the microfluidic system consisted of a top polydimethylsiloxane (PDMS) substrate (Layer A), an etching-treated indium-tin-oxide (ITO) glass substrate (Layer B), an adhesive tape with microfabricated microchannels (Layer C, thickness: 100 μm), and a bottom ITO glass substrate with a coating layer of photoconductive material (encompassing a 10-nm-thick molybdenum layer and a 1-μm-thick hydrogenated amorphous silicon layer) (Layer D). Structurally, the three through-holes were located in Layer A. Layer B contained three corresponding through-holes, allowing the connections of the three through-holes in the Layer A with the microchannels in Layer C. The T-shaped microchannel was located in Layer C.

### Microfabrication and experimental setup

The overall fabrication process was based on a computer-numerical-controlled (CNC) and a metal mould-punching fabrication process, a PDMS replica moulding, a thin-film technology using sputtering and plasma-enhanced chemical vapour deposition (PECVD), and a plasma oxidation-aided bonding process^36^. Briefly, the three components of the top PDMS layer (Layer A) [Fig. 1(b)] were fabricated by the combination of CNC machining and PDMS replica moulding. Briefly, three positive polymethylmethacrylate (PMMA) moulds for the three components were fabricated using a CNC miller (EGX-400, Roland Inc., Japan) with a 0.5 mm drill bit (rotational speed: 26,000 rpm). In the following replica moulding process, PDMS (Sylgard^®^ 184, Dow Corning, USA) was prepared by thoroughly mixing the PDMS prepolymer with a curing agent in a 10:1 ratio by weight. The mixture was then poured onto the fabricated PMMA moulds and cured at 70 °C for 1 hr. The cured PDMS parts were then obtained from a de-moulding process. For the preparation of the ITO glass substrate (Layer B) [Fig. 1(b)], the ITO on the glass was microfabricated through etching treatment using 2 N Hydrochloric Acid (Avantor™, PA, USA) solution, allowing the ITO material above the microchannel was preserved. After that, the three through-holes were mechanically drilled in the treated ITO glass (15 Ω, 0.7 mm; Ritek, Taiwan) using a driller (rotational speed: 26,000 rpm). For Layer C, a custom-made metal mould was used to create the hollow structure of the T-shaped microchannels in a double-sided adhesive tape (9009, 3 M, Taiwan) through a manually-punching process. For the bottom substrate (Layer D) [Fig. 1(b)], a 70-nm-thick ITO was first sputtered onto a cleaned dummy glass, followed by a thermal annealing process (240 °C, 60 min). A 10-nm-thick molybdenum metal layer was then sputtered onto the ITO layer to improve the adhesion between the fabricated ITO glass and the amorphous silicon layer to be deposited in the subsequent process. Next, a 1-μm-thick amorphous silicon layer was deposited onto the treated ITO glass through a PECVD process^36^.

In the following assembly process, Layer A (three through-hole components) was bonded with Layer B with the aid of O_2_ plasma surface treatment, followed by assembly with Layer D through the fabricated double-sided adhesive tape (Layer C) [Fig. 1(b)]. In operations, the loaded cell suspension sample was transported in the main microchannel using a suction-type syringe pump. To achieve the ODEP force-based cell manipulation^36^, a function generator was used to apply an alternating current (AC) voltage between the two ITO glass layers (Layer B and D) of the proposed system [Fig. 1(b)]. A commercial digital projector (PLC-XU350, SANYO, Japan) coupled with a computer was used to display controllable light images onto the photoconductive material (Layer D) to generate ODEP force on cells. In addition, a CCD-equipped microscope (Zoom 160, OPTEM, USA) was utilized to observe the manipulation of cells in the proposed system. The illustration of the overall experimental setup is schematically shown in Fig. 1(c) (a photograph of the experimental setup is provided as a supplementary figure: Fig. S1).

### Working principle of the ODEP microfluidic system for CTC purification

In this work, the combinations of ODEP force-based cell manipulation and laminar flow phenomenon in the proposed microfluidic system were utilized to separate the desired cancer cells from the leukocytes under the guidance of fluorescent microscopic observation. The overall operating procedures are illustrated in Fig. 2. Briefly, all cells including a human prostate cancer cell line (PC-3) and leukocytes in a sample were first stained with fluorescent dyes for EpCAM surface marker-positive cancer cells (green dot images), CD45 surface marker-positive leukocytes (red dot images), and Hoechst dye-positive nucleated cells (blue dot images). After that, the treated cell suspension sample was loaded in the microfluidic system and transported through the main microchannel in an inherent laminar flow pattern. Meanwhile, fluorescent microscopic observation was conducted at the CTC isolation zone to detect the cancer cells (green dot images) [Fig. 2-(I)]. Once the green dot images were observed, the cell suspension flow was suspended [Fig. 2-(II)] for further cancer cell identification and positioning. In the subsequent steps, the optical filters in the fluorescent microscopy were switched to observe the red dot images (leukocytes) [Fig. 2-(III)] and blue dot images (all nucleated cells) [Fig. 2-(IV)]. Through this contradistinction, the target cancer cells (e.g., the EpCAM marker and Hoechst dye-positive and CD45 marker-negative cells) in the cell mixture were then precisely positioned under light field microscopy imaging. The ODEP force-based cell manipulation was then performed in which hollow circular light images (OD: 120 μm; ID: 40 μm) were used to enclose the target cancer cells [Fig. 2-(V)]. Meanwhile, a moving long rectangular light bar (bar length: 1000 μm; bar width: 150 μm; moving velocity: 100 μm s^−1^) was utilized to sweep the all unenclosed leukocytes to one side of main microchannel, leaving the enclosed cancer cells at the same positions illustrated in Fig. 2(V–VII). This step was soon followed by moving the hollow circular light images (moving velocity: 50 μm s^−1^) with cancer cells enclosed to the side microchannel for collection [Fig. 2-(VII)-(VIII)]. Through these processes, the target cancer cells can be effectively separated from the leukocytes. The isolated cancer cells in the side microchannel were then transported to the site near the through-hole [Fig. 1(a)] for cancer cell collection using another moving rectangular light bar (bar length: 1000 μm; bar width: 150 μm; moving velocity: 100 μm s^−1^) [Fig. 2-(IX,X)]. After repeating the above processes, the cancer cells gathered in the side microchannel were finally obtained using a suction-type syringe pump.

### Optimization of T-shaped microchannel design and sample flow rate via the Computational Fluid Dynamics (CFD)-based simulation and experimental validations

In this study, CFD-based simulations, and microscopic observations were carried out to determine the appropriate side microchannel width, and sample flow rate that have less extent of contamination from the leukocytes in the sample flow to the side microchannel. Simulations were carried out using the COMSOL Multiphysics v3.5 commercial package. All simulations were performed in 2D. Sample flow models solved the incompressible Navier − Stokes equation, assuming an incompressible and laminar fluid, and no-slip boundary conditions to all microchannel walls. The total number of mesh elements was 14,581 (side microchannel width: 400 μm), 19,107 (side microchannel width: 700 μm) and 22,010 (side microchannel width: 1000 μm). For the experimental validations, 10^4 ^normal human leucocytes, and 500 PC-3 cells were mixed in a 10 μl sucrose solution. The cell suspension was then transported in the main microchannel under three different flow rate conditions (1.0, 2.5, and 5.0 μl min^−1^) (given side microchannel width: 400 μm). The travel distance of a cell from the main to side microchannel was observed microscopically.

### Working principle and optimal operating conditions for ODEP force-based cell manipulation

The utilization of ODEP force for microparticle manipulation was first proposed by Ming C. Wu^40^. In operation, briefly, an AC voltage is first applied between the two ITO glass substrates [Layer B and D; Fig. 1(b)] to produce a uniform electric field. In the electric field, microparticles (e.g., cells) become electrically polarised. When the amorphous silicon layer, a photoconductive material [the Layer D; Fig. 1(b)], is illuminated, the projected light can excite its electron-hole pairs and thus significantly decrease the electrical impedance of the illuminated area. This phenomenon causes the applied voltage to drop across the liquid layer inside the illuminated area, consequently creating a non-uniform electric field in the light-patterned regions. In the ODEP force-based cell manipulation, the generated non-uniform electric field is used to manipulate the electrically polarised cells^36^. Based on this principle, the cells can be simply manipulated by the optical images illuminated on the photoconductive layer.

In this study, the appropriate conditions under which to generate the ODEP manipulation force on cancer cells (e.g., PC-3 cells) and leukocytes were experimentally investigated based on the method described previously^36^. In a steady state, briefly, the ODEP manipulation force of a cell is balanced by its viscous drag of fluid. Therefore, the hydrodynamic drag force of a moving cell could be used to evaluate the net ODEP manipulation force of a cell according to Stokes’ law (equation 1). At a given cell size and viscosity of the surrounding solution, the ODEP manipulation force of a cell generated under a specific operating condition can be estimated from the terminal velocity of the cell. The terminal velocity is defined as the maximum manipulation velocity of a moving light image that can manipulate a cell^36^. For the cell species (leukocytes, and PC-3 cells) studied in this work, the maximum manipulation velocity under the ODEP operating conditions [(leukocytes: voltage magnitude range: 2–10 V, voltage frequency: 100 kHz, bandwidth of light bar: 150, 200, and 250 μm) (PC-3 cells: voltage magnitude range: 2–10 V, voltage frequency: 100 kHz, bandwidth of hollow circular light images (ID: 40 μm): 20, 40, and 60 μm)] explored was then experimentally quantified. Through these fundamental investigations, the optimal operating conditions for the ODEP force-based cell manipulation were determined.

### Immunofluorescence cell staining

To identify the cancer cells and leucocytes in the CTC isolation zone [Fig. 2-(II)], immunofluorescence staining and fluorescent microscopic observation were performed. In this study, the EpCAM and CD45 surface markers were used to identify the PC-3 cells^36^ and leukocytes, respectively. In addition, Hoechst dye was used to recognize the nucleated cells. All the assays were conducted according to the manufacturer’s instructions, as described previously^36^. Then, microscopic observations were conducted using a fluorescent microscope (Zess Axoviet 200 M). In these observations, the cells that were anti-CD45 dye negative, anti-EpCAM dye positive (green dots), and Hoechst dye positive (blue dots) were identified as the cancer cells. Conversely, the cell images that were anti-CD45 dye positive (red dots), anti-EpCAM dye negative, and Hoechst dye positive (blue dots) were identified as leukocytes.

### The performance evaluation of the proposed CTC isolation method

This study was approved by the Institutional Review Board of the Chang Gung Memorial Hospital, and informed consent was obtained from all blood sample donors (Approval ID: 104-8584C). All the methods were carried out in accordance with the relevant guidelines. Briefly, blood samples (8 ml each) were obtained from 3 healthy donors. A cell suspension sample was prepared by spiking PC-3 cancer cells (500 cells) (as a CTC model) into a whole blood sample (8 ml). The resulting average cancer cell number per millilitre of blood was 62.5 that mimics the rarity of CTCs in a blood sample. This was followed by a negative selection-based CTC isolation process to isolate the cancer cells. In operation, briefly, the erythrocytes and leukocytes in the blood sample were depleted using Ficoll-Paque^TM^ PLUS (GE Healthcare, Uppsala, Sweden) and Human CD45 depletion kits (STEMCELL^TM^, Vancouver, Canada), respectively. This was further processed by the ODEP-based CTC isolation scheme described in Fig. 2. Briefly, before these operations, the cell pellets obtained from the negative selection-based CTC isolation method were stained with immunofluorescent dyes, and then re-suspended in 30 μl of 250 mM sucrose solution (solution conductivity: 3.4 μS cm^−1^)^36^. In the sucrose solution, 0.5% (w/v) of bovine serum albumin (BSA, Sigma, St. Louis, Missouri, USA) was added to eliminate the electrostatic attraction between the cells and the inner surface of the microchannels, facilitating the subsequent ODEP force-based cell manipulation^35^. After the ODEP-based CTC isolation process (Fig. 2), the cell purity of cancer cells harvested was examined microscopically. In addition, the cancer cell recovery rate was also evaluated by the equation: [(the counted number of cancer cells harvested)/(the number of cancer cells spiked in a blood sample)*100%]. In order to evaluate the minimum number of cancer cells in blood that can be isolated, moreover, cell suspension samples with different cancer cell densities (60, 40, 20, 15, and 10 cells ml^−1^) were prepared, and treated with the ODEP-based CTC isolation as previously described. To demonstrate the use of the proposed ODEP microfluidic system for the isolation of real CTCs from cancer patient’s blood, an additional experiment was performed. Briefly, 8 ml of blood sample was obtained from a metastatic oral cancer patient, and followed by the two-step CTC isolation process as aforementioned. The cell purity of CTCs harvested was examined microscopically.

### The impact of leukocyte contamination on the analysis of the anticancer drug-resistance gene expression of cancer cells

Briefly, blood samples (16 ml each) were obtained from 3 healthy donors. For each, 1,000 PC-3 cells were spiked into the blood sample (average cancer cell number per millilitre blood: 62.5). One half of the sample (8 ml) was treated with a negative selection-based CTC isolation method to isolate the cancer cells as aforementioned. In addition to these treatments, the other half of the sample (8 ml) was further processed by the ODEP-based CTC isolation scheme described in Fig. 2. After the CTC isolation using the two abovementioned methods, the obtained cells were analysed for their gene expression [e.g., the mRNA levels of EpCAM, CD45, MRP4, MRP5 and GAPDH (a house-keeping gene)] based on a commonly used method^46^. First, the analysis of the mRNA levels of EpCAM, and CD45 of cells obtained through the two CTC isolation methods was to compare the purity of CTCs harvested. In addition, the expression analysis of two drug resistance genes (i.e., MRP4, and MRP5) of the cells was to examine to what extent the analysis of cancer cells’ anticancer drug-resistance gene expression levels can be influenced by the existence of the background leukocytes in the sample obtained. Briefly, the cells were subjected to RNA extraction (Dynabeads^®^ mRNA DIRECT^TM^ Micro Purification Kit, Thermo Fisher Scientific, MA USA), followed by complementary DNA (cDNA) synthesis using the TaqMan^®^ PreAmp Cells-to-CT™ Kit (Thermo Fisher Scientific, MA USA). The mRNA levels were then quantitatively determined using a StepOne™ Real-Time PCR System (Thermo Fisher Scientific, MA USA)^47^.

## Conclusions

The analysis of the anticancer drug-resistance gene expression of cancer patient’s CTCs could open a new horizon for achieving personalized chemotherapy. To accurately conduct such analytical work, the CTCs isolated from the blood sample of a cancer patient must be pure to exclude the possible interference of leukocytes in the cell sample tested. Nevertheless, the current CTC isolation schemes have technical limitations in harvesting high-purity CTCs. To address this technical hurdle, we proposed an ODEP microfluidic system to further purify the CTCs after a conventional CTC isolation process. In the presented ODEP microfluidic system, the inherent laminar flow in a microchannel was used to transport a cell suspension sample, immunofluorescent cell imaging was utilized to identify and position the target cancer cells, and then ODEP force was exerted to precisely separate the cancer cells from the leukocytes. In this work, a microfluidic system with a T-shaped microchannel was designed and fabricated. CFD-based simulations and experimental validations were performed to determine the appropriate width of the side microchannel (400 μm) and working flow rate of sample flow (2.5 μl min^−1^) to avoid cell contamination from the sample flow. In addition, the optimal ODEP conditions for the manipulation of PC-3 cells and leukocytes were experimentally evaluated. Within the experimental conditions studied, the results revealed that the electric voltage of 8 V applied in the ODEP system was able to generate the highest ODEP manipulation force for both the PC-3 cell and leukocyte manipulations without cell aggregation. For the leukocyte manipulation, a rectangular light bar with 150 μm bandwidth and a moving velocity of 100 μm/s was able to simultaneously manipulate the live and dead leukocytes. For the PC-3 cell manipulation, similarly, hollow circular light images (fixed ID: 40 μm) with 40 μm bandwidth and a moving velocity of 50 μm/s were able to effectively manipulate the live and dead cancer cells. Based on the proposed CTC isolation scheme, the results demonstrated that the presented system could isolate CTCs with cell purity as high as 100% (cell recovery rate: 41.5%), beyond the levels currently possible using existing techniques. In the analysis of the gene expression levels of CTCs, therefore, this approach could exclude the interferences caused by leukocytes in a cell sample and accordingly contribute to higher analytical sensitivity, as demonstrated in this study. Overall, this study has presented an ODEP-based microfluidic system capable of simply and effectively isolating a specific cell species from a cell mixture. In addition to CTC isolation, the presented system might also be found to be useful in any life science-related research in which the harvest of high-purity cells is required.

## Additional Information

**How to cite this article**: Chiu, T.-K. *et al*. Application of optically-induced-dielectrophoresis in microfluidic system for purification of circulating tumour cells for gene expression analysis- Cancer cell line model. *Sci. Rep.* **6**, 32851; doi: 10.1038/srep32851 (2016).

## Supplementary Material

Supplementary Video 1

Supplementary Video 2

Supplementary Video 3

Supplementary Video 4

Supplementary Video 5

Supplementary Information
