# Supplementary material for: Application of optically-induced-dielectrophoresis in microfluidic system for purification of circulating tumour cells for gene expression analysis- Cancer cell line model
Source: Sci Rep. 2016 Sep 9;6:32851. doi: 10.1038/srep32851 (PMC5016898; doi:10.1038/srep32851)
Supplement: Supplementary Information [file srep32851-s6.pdf]

A revised paper submitted to

Scientific Reports

Application of optically-induced-dielectrophoresis in microfluidic system for purification of circulating tumour cells for gene expression analysis- Cancer cell line model

Tzu-Keng Chiu[1], Wen-Pin Chou[2], Song-Bin Huang[2], Hung-Ming Wang[3], Yung-Chang Lin[3], Chia-Hsun Hsieh[1, 3*], Min-Hsien Wu[2,3*]

[1]Department of Chemical and Materials Engineering, Chang Gung University, Taoyuan City, 33302, Taiwan (R. O. C.)
[2]Graduate Institute of Biochemical and Biomedical Engineering, Chang Gung University, Taoyuan City, 33302, Taiwan (R. O. C.)
[3]Division of Haematology/Oncology, Department of Internal Medicine, Chang Gung Memorial Hospital, Taoyuan City, 33302, Taiwan (R. O. C.)

*Corresponding author (Circulating tumour cells): Dr Chia-Hsun Hsieh

E-mail: wisdom5000@gmail.com

Tel: +886-3-3281200 ext. 2517

Fax: +886-3-3281200 ext. 2362

*Corresponding author (Microfluidics and ODEP): Dr Min-Hsien Wu

E-mail: mhwu@mail.cgu.edu.tw

Tel: +886-3-2118800 ext. 3599

Fax: +886-3-2118668

Supplementary Figures

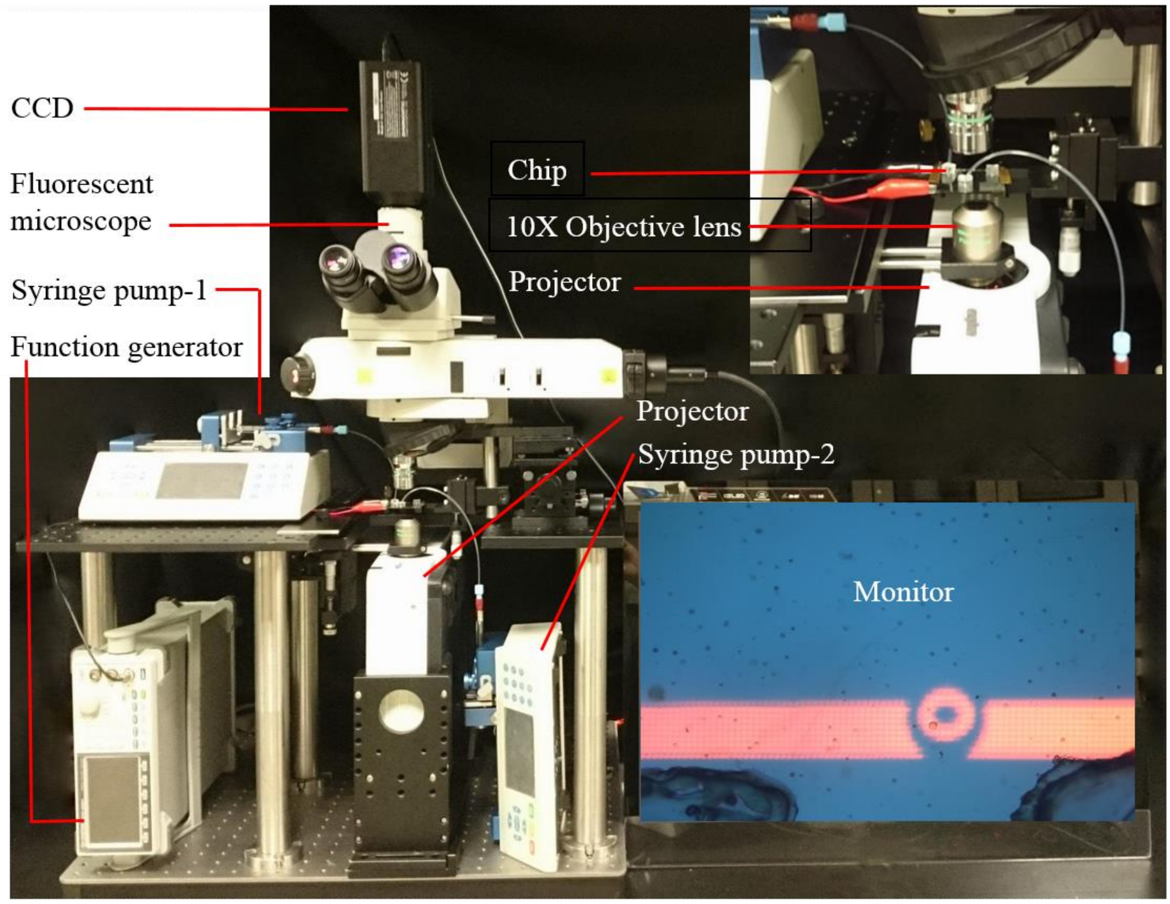

Fig. S1 Photograph of overall experimental setup

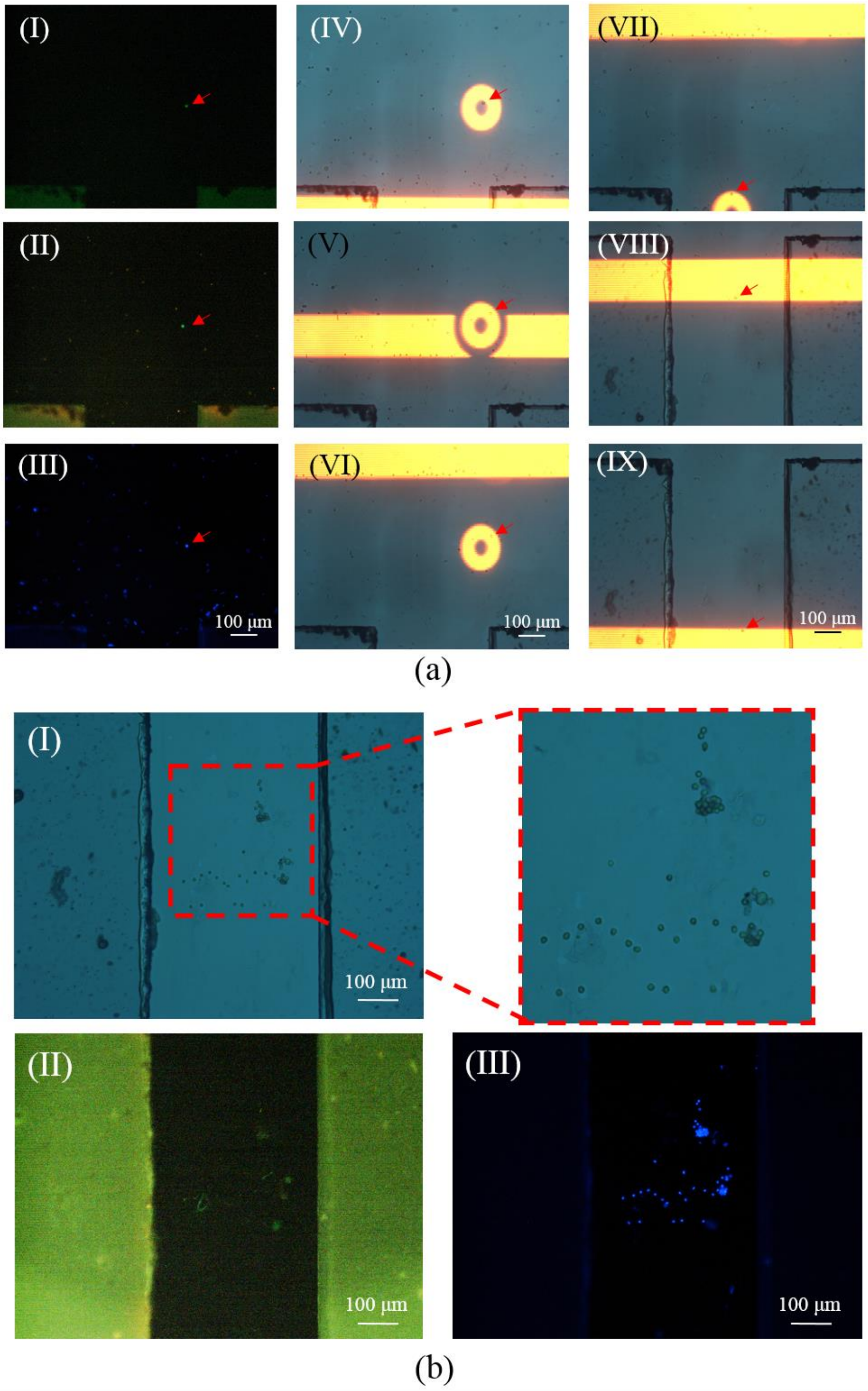

Fig. S2 (a) The overall CTC isolation and purification processes (Clinical sample test): (I) the cell suspension flow was temporarily suspended when a cancer cell (a green dot) was observed in the CTC isolation zone; (II)-(III) fluorescent microscopy operations were performed to observe the leukocytes (red dots), cancer cells (green dots), and

all nucleated cells (blue dots) for cancer cell positioning purposes; (IV) a hollow circular light image was used to enclose the target cancer cells, and a long rectangular light bar was used to manipulate the leukocytes; (V)-(VI) the long rectangular light bar was moved to sweep all unenclosed leukocytes to one side of the main microchannel, leaving the enclosed cancer cells at the same positions; (VII) the circular light image was moved to manipulate enclosed the cancer cells to the side microchannel for collection; (VIII)-(IX) another moving rectangular light bar was used to transport the cancer cells collected to a site near the through-hole for harvesting, (b) (I) a light field microscopy was used to observe the cancer cells harvested at the side microchannel, (II)-(III) immunofluorescent microscopic observations were performed to examine the purity of cancer cells [the leukocytes (red dots), cancer cells (green dots), and all nucleated cells (blue dots)]. (Two video clips are provided as the 4th and 5th video clips.)

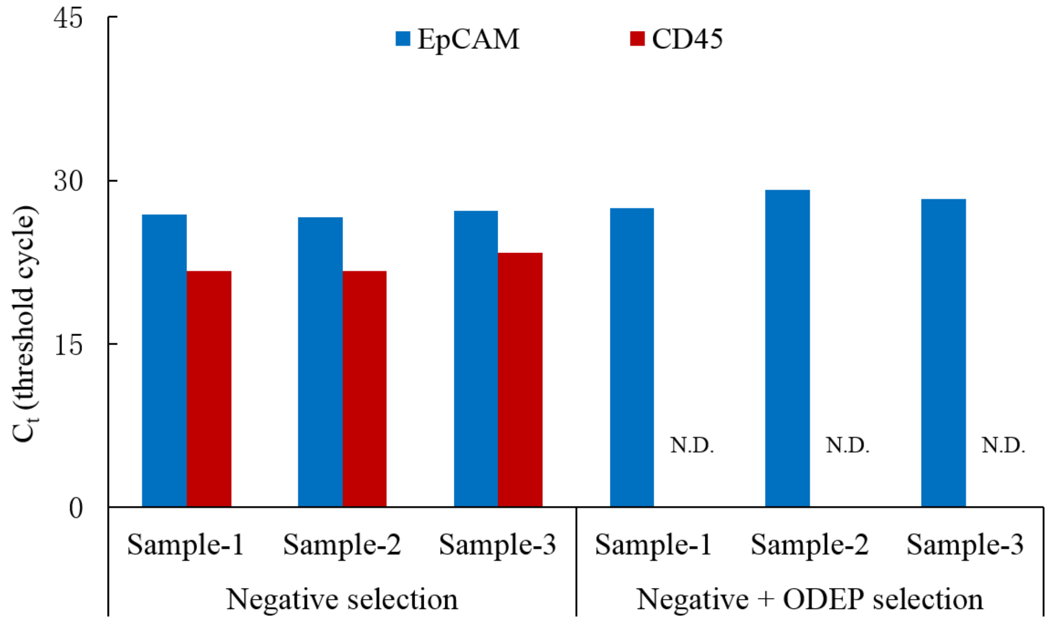

Fig. S3 Evaluations of the EpCAM and CD45 gene expression levels of cancer cells isolated from the two CTC isolation schemes studied (namely, a negative selection-based CTC isolation method and the same method with an additional ODEP-based CTC isolation process). Three individual samples were tested, and the gene expression levels were expressed as Ct, the threshold cycle, representative of the relative concentration of target nucleic acids in a PCR reaction (its level is inversely proportional to the quantity of target nucleic acids in a sample).

Video legend

1st video clip: The CTC isolation and purification processes (Cancer cell line model)

2nd video clip: The CTC isolation and purification processes (Cancer cell line model)

3rd video clip: The CTC isolation and purification processes (Cancer cell line model)

4th video clip: The CTC isolation and purification processes (Clinical sample test)

5th video clip: The CTC isolation and purification processes (Clinical sample test)
